# Systematic analysis of hip-preserving treatment for early osteonecrosis of the femoral head from the perspective of bibliometrics (2010–2023)

**DOI:** 10.1186/s13018-023-04435-8

**Published:** 2023-12-13

**Authors:** Tingyu Wu, Yaping Jiang, Hua Tian, Weipeng Shi, Yingzhen Wang, Tao Li

**Affiliations:** 1 Department of Joint Surgery, The Affiliated Hospital of Qingdao University, No. 59, Haier Road, Qingdao, 266000 China; 2 Department of Oral Implantology, The Affiliated Hospital of Qingdao University, Qingdao, 266003 China; 3 Department of Neurological Rehabilitation, Qingdao Special Servicemen Recuperation Center of PLA Navy, Qingdao, 266000 China

**Keywords:** Osteonecrosis of the femoral head, Hip-preserving treatment, Hotspot, Bibliometric, VOSviewer, CiteSpace

## Abstract

**Background:**

Osteonecrosis of the femoral head (ONFH) is a serious condition that causes bone tissue death, femoral head collapse, and hip joint destruction. Early intervention through hip-preserving treatment is crucial to slow down disease progression, preserve hip joint function, and improve the quality of life of patients. We analyzed the knowledge map, research gaps, and future research directions in the field of hip-preserving treatment for early ONFH.

**Methods:**

All publications related to hip-preserving treatment for early ONFH published between 2010 and 2023 were identified from the Web of Science Core Collection and analyzed using VOSviewer 1.6.19, CiteSpace 6.2.R2, and Scimago Graphica 1.0.35.

**Results:**

In total, 234 articles were analyzed. The results showed an exponential growth trend in the number of publications related to hip-preserving treatment for early ONFH in the past decade. China and the USA were the main contributors. *International Orthopaedics* published the most papers in this field, whereas *Bone and Joint Surgery-American Volume* had the highest average citation count per article. Several stable research topics were noted in this field, including core decompression (CD), osteotomy, bone transplantation in hip-preserving surgery, and cell therapy, which have become research hotspots in hip-preserving treatment.

**Conclusions:**

Hip-preserving treatment for early ONFH has received increasing attention, and research in this field is expected to grow. Stable research topics include core decompression (CD), osteotomy, bone transplantation, and cell therapy. Future research is predicted to focus on cell therapy and combination therapy, resulting in an increasing number of publications on hip-preserving treatment for early ONFH.

## Introduction

Osteonecrosis of the femoral head (ONFH) is a debilitating condition with significant global implications. This disorder afflicts millions of individuals worldwide, causing severe pain, reduced mobility, and eventual joint collapse [1]. In the USA alone, it is estimated that over 20,000 new cases of ONFH are diagnosed annually, underscoring its substantial impact [2]. Notably, the prevalence of ONFH continues to rise on a global scale, with China reporting a staggering 8.12 million nontraumatic osteonecrosis cases, posing a considerable challenge for orthopedic surgeons in the region [3].

The pathogenesis of ONFH has been a subject of ongoing debate, with various theories proposed. It is believed to involve alterations in coagulation mechanisms, fat embolism, disturbances in stem cell differentiation, cell apoptosis, osteoporosis, and genetic susceptibility [4]. However, the exact pathogenesis of ONFH remains elusive. In the early stages of ONFH, tissue damage occurs due to ischemia and hypoxia, resulting in the death of bone marrow cells and structural deterioration within the bone. As the disease progresses, changes in the shape and structure of the femoral head culminate in its collapse [5].

The collapse of the femoral head severely compromises hip joint stability and function, leading to secondary hip degeneration, excruciating pain, limited mobility, abnormal gait, and a range of other distressing symptoms [6]. Given the progressive nature of ONFH, timely intervention with hip-preserving treatments during the early stages is of paramount importance. These interventions are aimed at slowing disease progression, preserving hip joint function, reducing pain, and ultimately enhancing the quality of life for affected individuals [7]. Although total hip replacement (THA) has traditionally been considered the preferred treatment for advanced stages of ONFH, there is growing interest in hip-preserving treatment, particularly for patients in the early stages of ONFH [8]. Despite the lack of consensus on the optimal treatment of early ONFH, several studies have suggested that stem cell therapy may be the most effective approach for preserving hip joint function [9].

Bibliometrics is crucial in medicine, addressing the limitations of literature reviews. It quantitatively assesses research domains using math and stats to reveal trends and predict hotspots. Although some bibliometric analyses have been conducted in the field of ONFH [10–14], the global research trends in ONFH have not received sufficient attention. A recent study in the field of hip-preserving treatment for ONFH examined 10,334 ONFH patients who underwent hip-preserving surgery between 2010 and 2019 [15]. This study focused on changes in hip-preserving surgery trends in the USA during that period. Concurrently, a bibliometric analysis of surgical procedures for hip joint preservation for ONFH suggests that global research in this area has increased, with the USA leading the way, and “pathophysiology” and “basic research” potentially emerging as the next hot topics [16]. In this bibliometric study, our objective was to provide a comprehensive overview of the current research on hip-preserving treatment for early ONFH, encompassing both surgical and non-surgical approaches. Based on studies published between 2010 and 2023, we identified the most popular and influential authors, journals, countries and assessed co-citation network in this field. Additionally, we determined the research gaps and proposed future research directions in this field. Our results offer insights into the research needs and clinical implications of hip-preserving treatment for early ONFH, contributing to the development of effective treatments for this condition.

## Methods

### Search strategies and data collection

For a reliable bibliometric analysis, we chose the Web of Science Core Collection, specifically the Science Citation Index Expanded (SCI-EXPANDED) and Social Sciences Citation Index (SSCI), known for its authority and coverage [17]. The following search terms were used: TS (Topics) = (((Femur Head Necrosis) OR (Femur Head Necroses) OR (Osteonecrosis of the Femoral Head) OR (Head Necrosis, Femur) OR (Necrosis, Femur Head) OR (Aseptic Necrosis of Femur Head) OR (Necrosis, Aseptic, of Femur Head) OR (Necrosis, Avascular, of Femur Head) OR (Ischemic Necrosis of Femoral Head) OR (Femoral Head, Avascular Necrosis Of) OR (Avascular Necrosis of Femoral Head, Primary) OR (Avascular Necrosis of Femur Head)) AND ((hip preservation) OR (hip preserving))). We did not restrict language and focused on studies and reviews from January 1, 2010, to July 1, 2023.

**Fig. 1:**
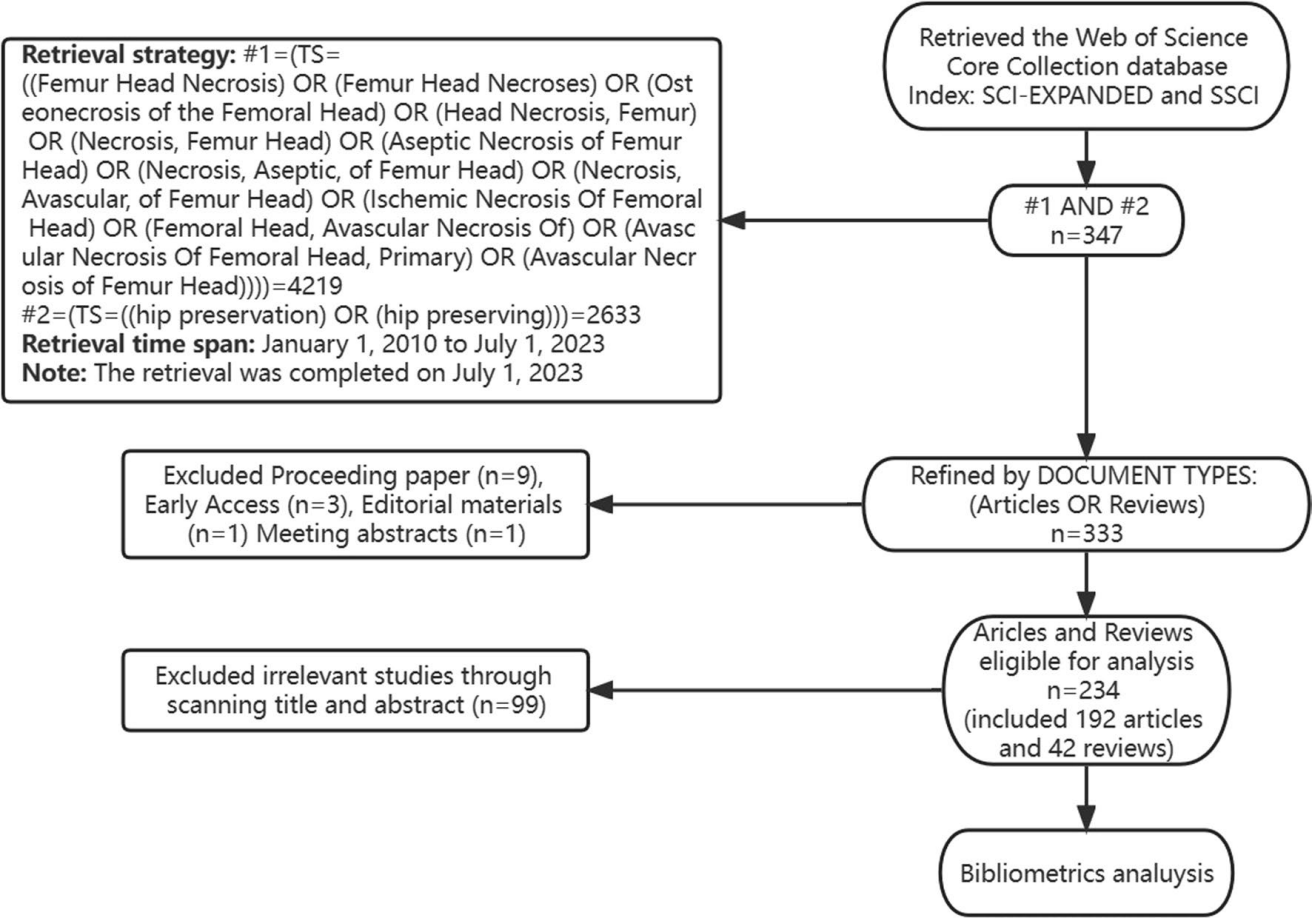
Flowchart of literature screening in this study

In bibliometric research, ethical concerns like data privacy and copyright are crucial. Researchers must respect author privacy, follow copyright laws, transparently disclose data sources, cite work honestly, reveal conflicts of interest, and undergo ethical reviews. These actions ensure research legality, integrity, and social responsibility, enhancing scientific inquiry’s credibility and sustainability. This study relies on existing open databases and involves two authors, one for data collection and the other for quality control, ensuring evidence reliability. No animal or human testing or novel materials were used. In total, 234 articles were included in the analysis. Figure 1 presents the article selection process.

### Bibliometric analysis

Bibliometrics is a field that studies academic publications using statistical and mathematical methods. It analyzes aspects like quantity, quality, citations, authors, and topics to provide insights into research trends, impact, collaborations, and hotspots [18]. Established in 1969, bibliometrics has become crucial for scientific research and assessment, with broad applications across various fields, thanks to technology and information management advancements [19].

For a thorough analysis of publications on early ONFH hip-preserving treatment, we utilized three bibliometric tools: VOSviewer (1.6.19 edition), CiteSpace (6.2.R2 Advanced edition), and Scimago Graphica (1.0.35 edition). CiteSpace is a powerful tool for creating knowledge maps from literature, helping to uncover knowledge structures, research hotspots, and collaboration networks. It offers functions like co-authorship network analysis and topic evolution analysis, aiding in hotspot identification [20]. VOSviewer, another bibliometric tool, transforms a large volume of literature into knowledge maps. It visualizes citation relationships, authors, institutions, and topics, providing insights into knowledge structures and research hotspots in academic fields [21].

## Results and discussion

### Publication outputs and trends

This study included 234 articles from 1070 authors affiliated with 315 organizations across 30 countries. These articles were published in 94 journals and received 4383 citations from 1090 journals.

**Fig. 2:**
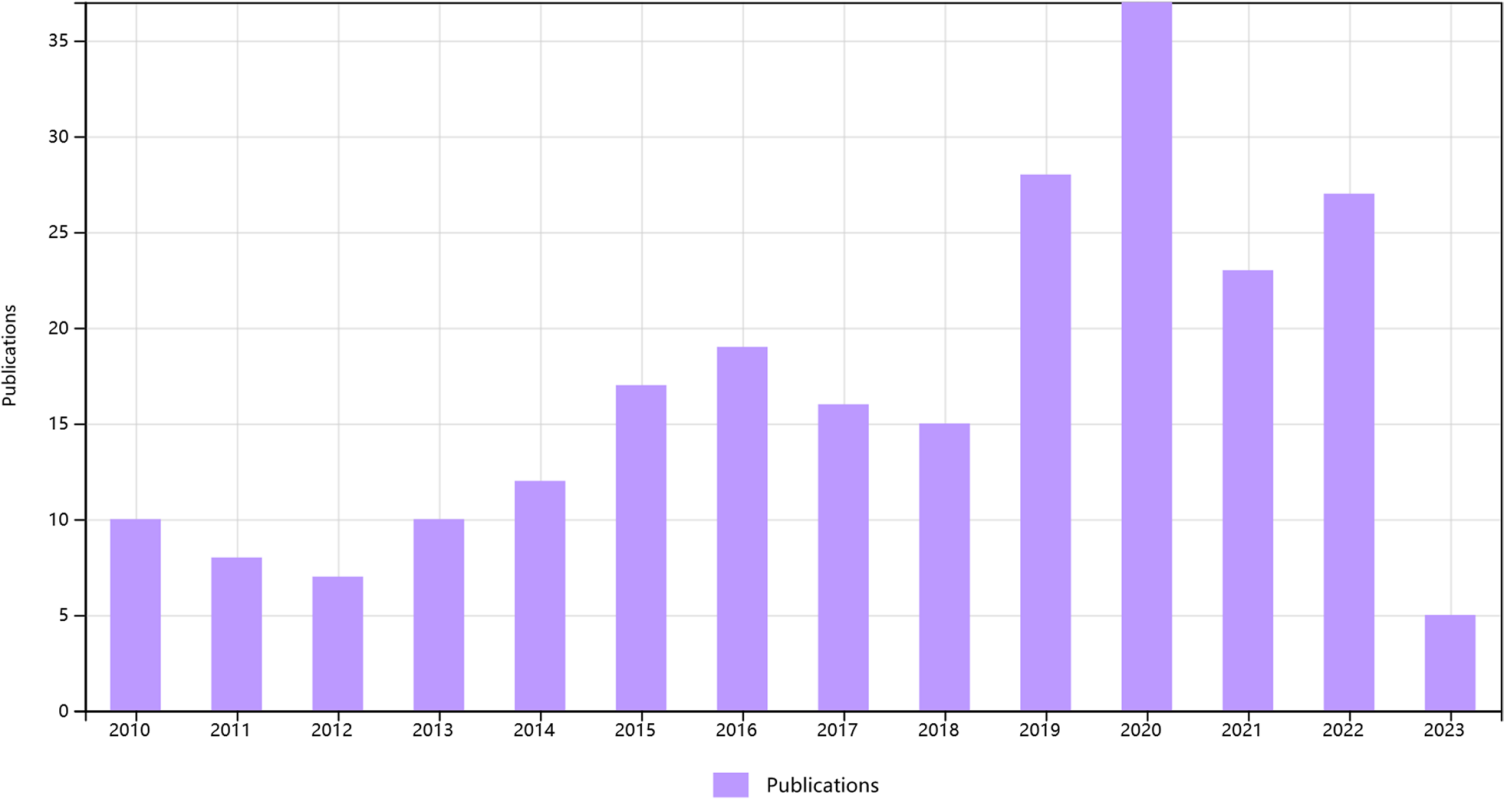
Distribution of publications from 2010 to July 2023

Figure 2 presents the temporal distribution of articles published in the field of hip-preserving treatment for early ONFH. Overall, there has been an increase in the number of publications in this field. In particular, the number of publications has exceeded 20 per year since 2019, indicating increasing attention from orthopedic surgeons in recent years. These findings suggest that hip-preserving treatment methods have garnered widespread international attention and hold promise for improving the treatment and quality of life for ONFH patients.

### Co-authorship analysis

#### Core authors

In 1926, Lotka [22] proposed Lotka’s law, stating that over 60% of authors write just one paper. Later, in 1963, Price et al. [23] demonstrated that a small group of highly productive authors, comprising approximately the square root of the total number of authors (Formula 1), write almost half of the papers on a given topic. Formula 2 calculates the minimum publications needed for core contributor status in a field.

1 $$\mathop \sum \limits_{m + 1}^{I} n\left( x \right) = \sqrt N$$

2 $$m = 0.749 \times \sqrt {n_{\max } }$$

where *n(x)* represents the number of authors who have written *x* papers, *N* is the total number of authors, *I* = *n*_max_ is the number of papers published by the most productive author in the field, and *m* is the minimum number of publications required to be considered a core author. Using VOSviewer analysis, we found that core authors in this field had published three or more papers, totaling 85 authors who contributed 131 papers, about 60% of the publication output. This suggests a stable a group of collaborating authors in the field of hip-preserving treatment for early ONFH. Table 1 lists the top five core contributors in this field.

**Table 1:** Most important authors in the field of hip-preserving treatment for early ONFH between 2010 and 2023

Rank	Author	Documents	Citations	Average citation/publication
1	He, W	15	83	5.53
2	Zhao, D	10	320	32
3	Sun, W	10	217	21.7
4	Mont, M	8	855	106.86
5	Wang, B	7	305	43.57

As shown in Table 1, He W. from the First Affiliated Hospital of Guangzhou University of Chinese Medicine contributed the most papers. Mont M. from Lenox Hill Hospital at Northwell Health was the most highly cited author. Notably, both scholars have an interest in cell therapy, as evidenced by their published papers [24, 25]. The papers written by He W. mainly focus on traditional Chinese medicine [26], vascularized bone graft (VBG) [27], and minimally invasive hip preservation surgery using tantalum rods [28], whereas those by Mont M. focus on non-vascularized bone graft (NVBG) [29].

This research highlights the significant contributions of two key researchers, He W. and Mont M., in the field of hip-preserving treatment for ONFH. He W.’s extensive publication record demonstrates his ongoing research and publishing activity in this field, while Mont M.’s remarkably high citation count underscores the academic impact of his research. Both scholars have explored diverse areas, including traditional Chinese medicine, bone grafting techniques, and cell therapy, offering rich research avenues and strategies for improving hip treatment in the future. This also underscores the importance of cell therapy in osteonecrosis treatment, paving the way for new prospects in clinical practice and patient care.

#### Core journals

In 1934, Samuel C. Bradford introduced Bradford’s law, highlighting the importance of core journals in various subject areas, which become more pronounced over time [30]. Based on Bradford’s journal classification, journals can be categorized into core, related, and peripheral zones, with their numbers following a ratio of *n*1:*n*2:*n*3 = 1:*a*:*a*^2^, where *n*1, *n*2, and *n*3 represent the numbers of journals in the core, related, and peripheral zones, respectively, whereas *a* represents the Bradford coefficient (*a* > 1). Following Bradford’s law, journals in this field were classified into three zones with an approximate ratio of 1:3:9 (or 1:3:3^2^; Table 2). This distribution pattern implies that the research papers in this field published from 2010 to July 2023 adhere to Bradford’s law, highlighting the concentration of most papers in core journals.

**Table 2:** Journal partition

Zone	Publication/journal	Number of journals	Number of publications
First zone	≥ 9	8	82
Second zone	3–8	19	76
Third zone	1–2	67	76

Table 3 displays the top 10 most prolific journals, of which *International Orthopaedics* (journal impact factor [JIF] = 2.7)*, BMC Musculoskeletal Disorders* (JIF = 2.3)*,* and *Journal of Orthopaedic Surgery and Research* (JIF = 2.6) have published 10 or more papers (17, 15, and 11 papers, respectively). Among the top 10 prolific journals (Table 3), seven are in the top 50% based on JIF rankings. The leading orthopedic journal is *Journal of Bone and Joint Surgery-American Volume*. This journal primarily features research articles, particularly focusing on postoperative imaging results and the risk of femoral head collapse in ONFH patients following hip-preserving treatment [31, 32].

**Table 3:** Top 10 journals in the field of hip-preserving treatment for early ONFH between 2010 and 2023

Rank	Sources	Publications	Citation	Average citation/publication
1	International Orthopaedics	17	406	23.88
2	BMC Musculoskeletal Disorders	15	178	11.87
3	Journal of Orthopaedic Surgery and Research	11	58	5.27
4	Orthopaedic Surgery	9	17	1.89
5	Archives of Orthopaedic and Trauma Surgery	9	176	19.56
6	Journal of Hip Preservation Surgery	7	24	3.43
7	Journal of Bone and Joint Surgery-American Volume	7	698	99.71
8	Clinical Orthopaedics and Related Research	7	351	50.14
9	Journal of Arthroplasty	6	126	21
10	Bone & Joint Journal	6	214	35.67

The results underscore the significant presence and influence of research related to hip-preserving treatment for early ONFH in academic journals. This includes prolific journals as well as highly cited ones. The research articles in these journals hold substantial academic value in advancing the treatment and research of early ONFH, providing a robust knowledge base and guiding directions.

#### Core countries

**Fig. 3:**
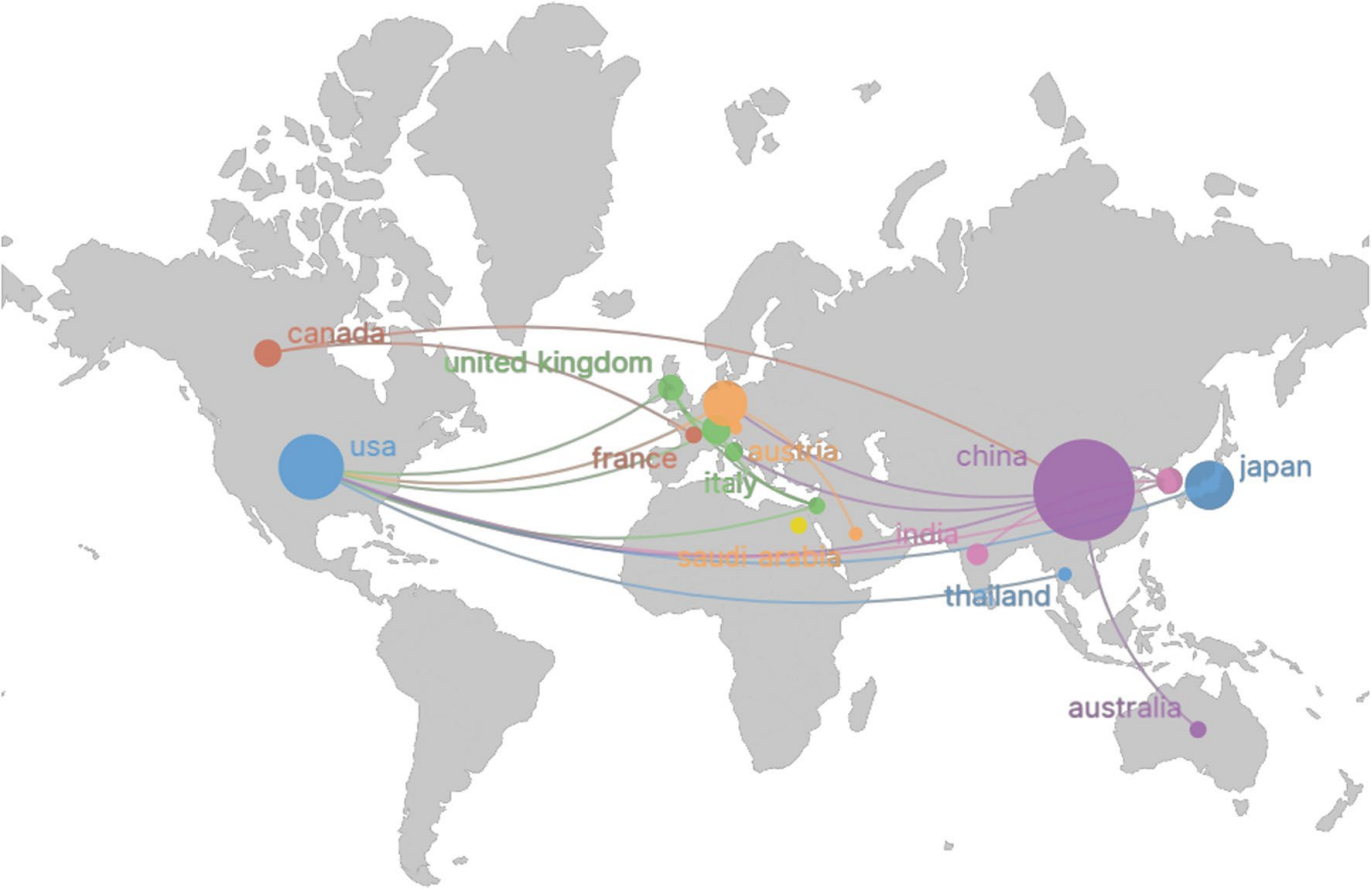
Co-occurrence of countries

Eligible articles, authored by investigators from 30 countries, were analyzed for collaborative patterns using VOSviewer and Scimago Graphica. Figure 3 displays collaborative publication counts between countries, with node size indicating each country’s publication count. The connections between nodes reflect collaborative publication frequency, with thicker lines indicating more frequent collaborations. Node colors denote distinct clusters, grouping countries with similar characteristics. Figure 3 reveals an imbalanced distribution of publishing countries in this field, indicating a notable “top-heavy” pattern where a few countries contribute to most of the papers.

Table 4 presents the top five countries in early ONFH hip-preserving treatment research. China leads with 108 publications, followed by the USA (45), Japan (25), Germany (22), and Switzerland (9). These countries contribute to 78.63% of the total publications. China has been the most prolific since 2010, but Chinese publications received comparatively fewer citations. In contrast, the USA boasts the highest average citation count per publication, indicating global academic influence.

**Table 4:** Top 5 countries in the field of hip-preserving treatment for early ONFH between 2010 and 2023

Rank	Country	Publications	Citations	Average citation/publication
1	China	108	1313	12.16
2	USA	45	1974	43.87
3	Japan	25	410	16.4
4	Germany	22	339	15.41
5	Switzerland	9	213	23.67

However, the concentration of research output in a few countries highlights the need for broader international collaboration and diverse perspectives in the field. Future research trends may involve enhanced global collaboration for comprehensive studies and improved treatment strategies. Furthermore, elevating research quality and impact, particularly in countries with high publication volumes like China, will be essential for advancing hip-preserving treatment for early ONFH.

### Co-occurrence analysis of keywords

**Fig. 4:**
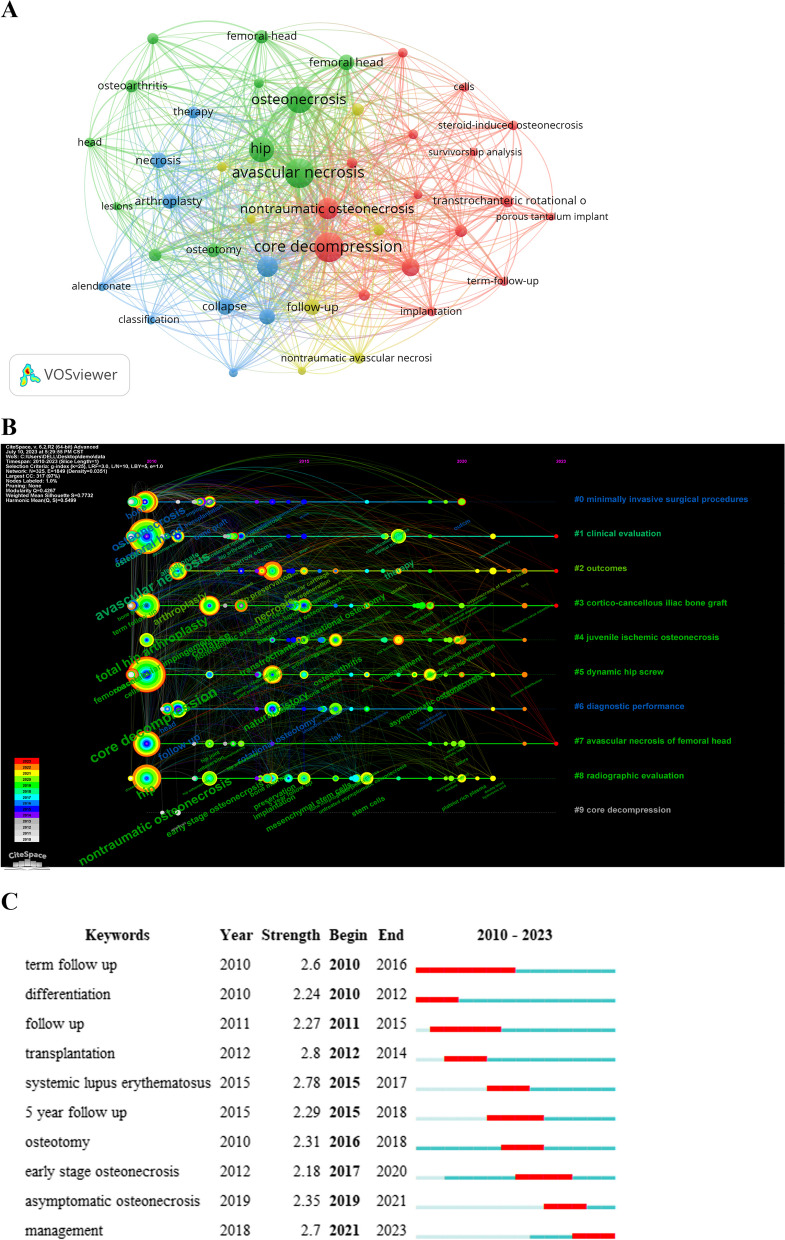
Keywords in the field of hip-preserving treatment for early ONFH. **A** Co-occurrence of high-frequency keywords; **B** The timeline view of high-frequency keywords; **C** Top 10 keywords with the strongest citation bursts

The study applied Formula 2 to identify high-frequency keywords (appearing at least eight times in 234 publications) and conducted visualization analysis using VOSviewer as shown in Fig. 4A. Table 5 lists keywords with frequencies exceeding 20. Figure 4A and Table 5 reveal that, apart from core keywords in the search terms, “core decompression” and “osteotomy” were high-frequency terms, emphasizing that the focus on hip-preserving treatment for early ONFH was primarily on these two methods.

**Table 5:** Top 15 keywords in the field of hip-preserving treatment for early ONFH between 2010 and 2023

Rank	Keywords	Frequency	Total link strength
1	Core decompression	101	311
2	Avascular necrosis	101	305
3	Osteonecrosis	84	230
4	Hip	74	207
5	Nontraumatic osteonecrosis	55	173
6	Osteonecrosis of the femoral head	50	122
7	Collapse	31	112
8	Femoral head	29	98
9	Arthroplasty	27	96
10	Total hip-arthroplasty	36	96
11	Follow-up	28	89
12	Natural-history	28	86
13	Necrosis	30	83
14	Osteotomy	21	72
15	Femoral-head	22	58

Core decompression (CD) is the primary surgical treatment for early ONFH. It involves drilling into the affected area to alleviate bone pressure by breaking up necrotic and sclerotic tissue [33]. However, the traditional CD approach yields suboptimal results, with around 38% needing THA about 26 months [34]. Recent evidence suggests core decompression’s efficacy is, at best, comparable to other joint-preserving strategies and may be less successful than alternatives [35]. Combining CD with new adjuvant therapies, as the current approach, shows better postoperative outcomes [36, 37]. CD combined with bone marrow-derived cell therapies reduces pain and lowers the THA rate compared to isolated CD [38]. Bone marrow concentrate with CD is more effective before femoral head collapse, especially when Kerboul combined necrotic angles are < 250° [39]. Outcomes can be further enhanced by adding platelet-rich plasma to cultured bone marrow concentrates [40]. While platelet-rich plasma CD alleviates pain and enhances function, long-term femoral head outcomes do not show significantly improve [41]. Combining bone morphogenetic protein with CD can improves hip joint survival but lacks sufficient efficacy data [42]. Bisphosphonates with CD ease pain, delay femoral head collapse, and are a safe, effective method for early and intermediate ONFH hip preservation [43]. Furthermore, Wang et al. [44] developed an augmented reality-based navigation system for precise Kirschner wire placement during surgery, reducing associated injuries. This approach enhances the surgical efficiency and patient prognosis in early ONFH.

Another commonly researched hip preservation method is osteotomy, which shifts the necrotic area away from weight-bearing regions and repositions the intact femoral head portion for weight-bearing. Common osteotomy techniques encompass transtrochanteric anterior rotational osteotomy [45], various angled intertrochanteric osteotomies [46], and curved intertrochanteric varus osteotomy [47]. The study by Quaranta et al. [48] indicated that following osteotomy for ONFH, approximately one-third of patients underwent THA within 7 years, and factors contributing to osteotomy failure remained unclear. However, Osawa et al. [47] showed that curved intertrochanteric varus osteotomy in patients under 50 years offers similar hip function and greater satisfaction than THA, suggesting osteotomy’s promise for hip preservation despite increased blood loss and technical challenges, requiring skilled surgeons. Consideration of age-related risks, potential conversion to THA, and subsequent morbidity and mortality is crucial when planning such procedures.

These research hotspots are crucial for improving the treatment and prognosis of early ONFH patients. CD and hip osteotomy represent pivotal surgical approaches, offering a range of treatment options to personalize patient care, alleviate pain, delay hip discomfort, and reduce the need for THA. Simultaneously, the application of new adjuvant therapies such as bone marrow concentrates, platelet-rich plasma, and growth factors, along with the development of augmented reality navigation systems, provides novel avenues to enhance surgical efficiency and patient outcomes. These studies drive progress in the field of hip disease treatment, delivering better quality of life and clinical results for patients.

### Evolution analysis of keywords

We created a visual knowledge map using CiteSpace’s “Timeline” function to explore research trends in early ONFH hip-preserving treatment. This map divided the research field into 10 main clusters (Fig. 4B). From 2010 to 2023, hip-preserving treatment for early ONFH has remained a research focus, with minimally invasive surgery gaining significant attention from both patients and researchers. For instance, Zhang et al. [49] discovered that minimally invasive T-type fibular support offers several benefits over traditional iliac flap metastasis, including less pain, reduced bleeding, smaller trauma, and shorter duration, making it an ideal bone graft technique.

Cluster 2 suggests ongoing research into the clinical prognosis of hip preservation surgery, comparing various procedures and hip preservation surgery versus THA. Some proposed assessing outcomes using four indicators: pain, hip flexion range, walking distance, and X-ray image stability assessment [50]. Cluster 4 focuses on hip-preserving treatment for juvenile ischemic osteonecrosis. Kamiya et al. [51] used an immature mouse model and discovered that tocilizumab, an interleukin-6 receptor inhibitor, promotes chondrogenesis and increases bone volume. Clinical trials are anticipated to assess its potential in preventing femoral head deformity in children with this condition. Furthermore, clusters 9 and 10 show a shift away from traditional CD as a research hotspot, with a growing focus on stem cell therapy. Our previous study has explored the current trends in stem cell therapy for ONFH, highlighting opportunities and challenges in clinical applications and related research [9]. These research areas are important as they directly impact the quality of life and treatment outcomes for individuals with hip disorders. Understanding the clinical prognosis of different hip surgeries, particularly hip preservation surgery and stem cell therapy, helps doctors choose the most suitable treatment for patients. These studies not only influence patient well-being but also have the potential to advance clinical practices and enhance treatment success rates.

To better grasp the sudden emergence of research hotspots in early ONFH hip-preserving treatment, we performed a Burst analysis using CiteSpace (Fig. 4C). Burst analysis identifies rapid increases in specific words or phrases within a research area, aiding researchers in identifying development trends and hotspots in the field [52]. Figure 4C shows an even distribution of burst words over time in this research field, without any significant yearly spikes.

The most prominent burst word was “transplantation,” signifying its effectiveness in hip preservation treatment for ONFH with high cure rates, durability and low complications. The lightbulb technique, involving a cortical window at the femoral head-neck junction for tissue removal and bone grafting, has significantly improved ONFH treatment outcomes, especially in early stages before femoral head collapse [53]. The rise of management research as a 2021 hotspot indicates increasing interest in this field. Early ONFH lacks consensus and effective treatments, as it often presents mild symptoms and minor lesions, posing diagnostic and treatment challenges. Some studies show a higher rate of hip preservation surgery in young patients compared to older ones, yet joint replacement surgery remains more common than preservation surgery [54]. On the other hand, Migliorini et al. [55] found that, in hip preservation for ONFH, being male, having prolonged pre-treatment symptoms, higher Visual Analog Scale and lower Harris Hip Score were negative prognostic factors. Hence, researching management and treatment strategies for early ONFH to discover more effective, safer, and practical methods is of great clinical and scientific significance.

These trends underscore the pressing need for continual improvement and innovation in the treatment of early ONFH to enhance patient outcomes and safeguard their hip joints. Minimally invasive surgeries and novel treatment approaches hold the promise of providing patients with more effective and safer options, subject to further validation through clinical studies. Additionally, the emphasis on research into early ONFH treatment for children highlights the unique requirements of pediatric patients and offers new avenues for preventing long-term bone damage. The emergence of stem cell therapy as a potential treatment represents an innovative approach, albeit one that necessitates further research to establish its safety and effectiveness. In sum, these research trends are poised to drive further advancements and enhancements in the field of early ONFH treatment.

### Co-citation analysis

#### Cited journals

**Fig. 5:**
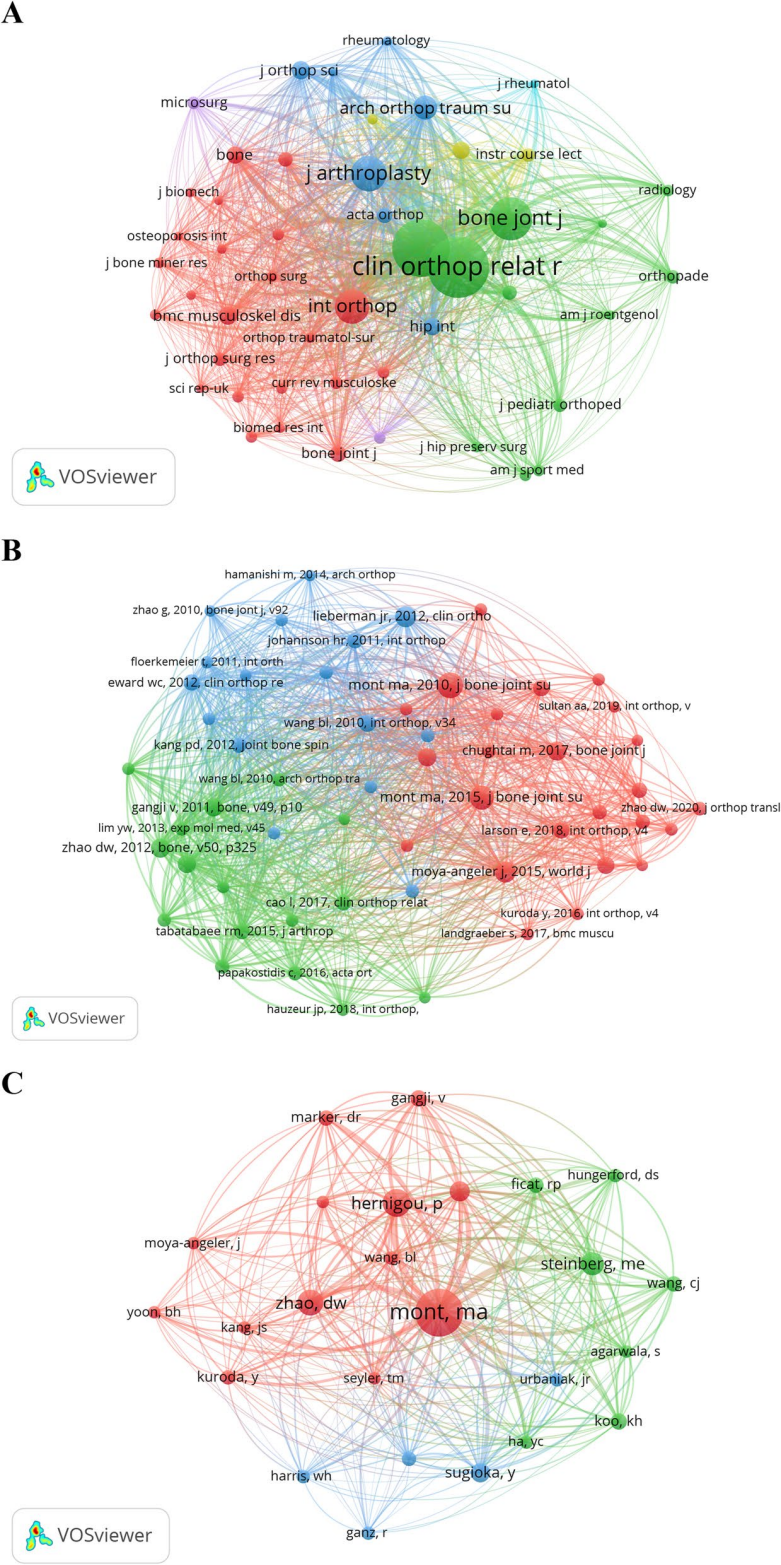
Co-citation analysis in the field of hip-preserving treatment for early ONFH. **A** Cited journals; **B** Cited references; **C** Cited authors

Co-citation analysis, conducted from 2010 to 2023 in the field of hip-preserving treatment for early ONFH, identified frequency cited papers and journals. Using VOSviewer with a minimum co-citation threshold of 30, 48 journals were analyzed, as shown in Fig. 5A. Figure 5A reveals a co-citation network of journals comprising six clusters, each represented by a distinct color. The top three frequently cited journals are *Clinical Orthopaedics and Related Research* (1309 citations), *Journal of Bone and Joint Surgery-American Volume* (1165 citations), and *Bone & Joint Journal* (645 citations). These journals are highly regarded in orthopedics and classified as excellent publications in JCR Q1 category.

In the six clusters, the red cluster mainly consists of orthopedic journals, emphasizing surgical ONFH treatments, serving as references for technical support. The green and dark blue clusters are linked to nuclear medicine and clinical orthopedics, concentrating on early imaging results and postoperative outcomes, providing foundational evidence for research. The yellow, purple, and light blue clusters pertain to rheumatology, focusing on ONFH causes, offering theoretical backing by analyzing etiology.

This reveals the research trends and key areas in this field. Through the identification of the most frequently cited papers and journals, it underscores the importance of academic journals in disseminating knowledge. The presence of six distinct clusters, represented by different colors, reflects the diversity of ONFH treatment research, spanning from orthopedic surgery to imaging, clinical studies, and rheumatology. This provides valuable insights for guiding future clinical practices, offering beneficial perspectives for enhancing early ONFH treatment, and fostering interdisciplinary collaboration.

#### Cited references

Using VOSviewer, we conducted co-citation analysis of literature in this field from 2010 to July 2023. Table 6 lists the top five most cited articles during this period. Notably, two of these articles were authored by the prolific researcher Michael A Mont focused on ONFH treatments. Mont’s work emphasizes the importance of joint-preserving surgical treatment for asymptomatic patients with moderate or large and/or lateral lesions, before femoral head collapse [56]. However, once femoral head collapse occurs, THA become the preferred option [57].

**Table 6:** Top 5 most important publications in the field of hip-preserving treatment for early ONFH between 2010 and 2023

Rank	Title	Year	Citations (2010–2023)
1	The natural history of untreated asymptomatic osteonecrosis of the femoral head: a systematic literature review	2010	49
2	Nontraumatic osteonecrosis of the femoral head: Where do we stand today? A ten-year update	2015	41
3	Which factors influence preservation of the osteonecrotic femoral head?	2012	32
4	Current concepts on osteonecrosis of the femoral head	2015	32
5	An evidence-based guide to the treatment of osteonecrosis of the femoral head	2017	27

Furthermore, we employed VOSviewer to construct a co-citation map of references, setting a minimum co-citation threshold of 10 citations. Notably, the references analyzed were cited after 2010. The resulting map encompassed 56 references for co-citation analysis (Fig. 5B). Figure 5B displays a co-citation network of highly cited references, categorized into three main clusters represented by different colors. The red cluster pertains to surgical hip preservation treatments, the green cluster comprises review articles, and the blue cluster is associated with non-surgical hip preservation research.

These findings highlight the significant contributions of experts in this field. The construction of co-citation networks aids in better understanding the relationships between different treatment methods and domains, offering valuable insights and collaboration opportunities for future research and clinical practice. Therefore, these results hold crucial significance for clinical decision-making and improving the treatment of early ONFH.

#### Cited authors

To pinpoint the top authors in this field from 2010 to 2023, we generated a co-citation map of primary authors using VOSviewer. We selected the top 25 most highly cited authors, setting a threshold of at least 30 co-citations for each author, from a pool of 3252 cited authors. The highly cited authors’ co-citation network is divided into three major clusters represented by different colors in Fig. 5C. The red cluster experts primarily specialize in fracture repair, encompassing diverse surgical techniques, biological aspects of fracture healing, and various facture treatment methods. They also possess extensive expertise in orthopedic emergency and trauma management. The green cluster authors mainly focus on joint replacement and orthopedic surgical treatment, emphasizing the evaluation of surgical techniques and treatment outcomes, including artificial joint replacement and arthroscopic surgery. The blue cluster authors concentrate on ONFH and hip joint replacement, emphasizing the evaluation of surgical techniques and treatment outcomes, including innovative surgical approaches and treatment plans.

These findings highlight the expertise in orthopedics from 2010 to 2023, covering fracture repair, joint replacement, and hip surgeries. The author clusters reflect the diversity and specialization within orthopedics, offering valuable insights for clinicians seeking specialized guidance and opportunities to advance orthopedic research and education. Furthermore, they contribute to enhancing clinical practice and ensuring optimal orthopedic care for patients.

## Diverse bibliometric perspectives in ONFH research

Researches on ONFH have diverse areas of focus. Our study specifically concentrates on the knowledge map, research gaps, and future research directions in the field of hip-preserving treatment for early ONFH. In contrast, other articles tend to emphasize different facets of ONFH research, such as global trends [11–13], hip-preserving surgical treatments [15, 16], and core treatment methods [10]. These articles encompass various time periods and countries, providing valuable insights within their respective domains. Furthermore, they underscore the diversity of research in the ONFH field, spanning from fundamental research to clinical treatment [14].

In future research endeavors, it may be worthwhile to amalgamate findings from these diverse research domains to construct a more comprehensive ONFH research framework. This integration could delve into early treatment methods and further explore the mechanisms underlying ONFH. By amalgamating research from these distinct areas, opportunities for enhancing early ONFH treatment strategies, improving patient quality of life, and alleviating the socioeconomic burden can be identified. This approach would facilitate a better understanding and management of this serious ailment.

## Strengths and limitations

This study employed VOSviewer and CiteSpace to assess the research on early ONFH hip-preserving treatment published in the last decade, identifying research trends. We also examined the relevance of Lotka’s, Price’s, and Bradford’s laws in bibliometrics for this filed. Notably, our analysis mainly focuses on research conducted after 2010 due to limited prior Chinese contributions in the early ONFH hip-preserving treatment methods. While Zhang et al. [16] have similar articles, they offer a more simplistic approach and a broader scope, making in-depth analysis and discussion challenging. Furthermore, they do not explore specific hip-preserving methods, whereas our study encompasses both surgical and non-surgical approaches in this field.

However, this study had certain limitations. Firstly, we selected journal articles from only the Web of Science Core Collection using SCI-EXPANDED and SSCI indexes; other databases were not searched, leading to an incomplete identification of the published articles. Secondly, we focused on post-2010 studies to stay current, but this might mean missing early influential research in hip-preserving treatment for early ONFH. This limitation could restrict our historical perspective. Thirdly, quantitative analysis requires researchers to have a comprehensive understanding of the field. A lack of such an understanding may lead to the introduction of subjective bias. Additionally, bibliometric tools rely on keywords, which can be subjective and may not capture emerging topics. Lastly, our study is based on past data, but the field is evolving, so our findings may become outdated, requiring regular updates for validity.

## Conclusions and future perspectives

The use of hip-preserving treatment for early ONFH has gained orthopedic specialists’ attention. Notable scholars exist, but more collaborative efforts are needed. *International Orthopaedics* publishes the most papers, whereas *Journal of Bone and Joint Surgery-American Volume* boasts the highest average citation count per article. Chinese scholars lead in paper publications, whereas American scholars have the highest citation recognition. Keyphrase co-occurrence and evolutionary analysis reveal stable research topics: CD, osteotomy, bone transplantation, and cell therapy are current hotspots. Notably, systemic lupus erythematosus, minimally invasive surgery, diagnosis, prognosis, and postoperative follow-up are frequently discussed.

While the statistical data for this study cover the period from 2010 to July 2023, it is anticipated that future trends in the field of hip-preserving treatment for early ONFH will involve a sustained growth in research interest driven by increasing patient demand. Potential developments may include deeper investigations into cell therapy, the emergence of innovative treatment methods and surgical techniques, and enhanced international collaboration to accelerate knowledge sharing and standardize treatment approaches. In essence, despite the study’s limited timeframe, the field is poised for continued expansion and holds promise for further innovations and collaborative opportunities.

In summary, this study highlights the growing interest in hip-preserving treatment for early ONFH. It identifies publication trends, collaboration opportunities, and research hotspots. Future research may focus on exploring cell therapy and innovative combinations with hip-preserving surgery in this evolving field.

## Data Availability

The data collected and analyzed in the article are from WOS, an open access database of scholarly articles, and are properly adopted and collected.

## Abbreviations

ONFH: Osteonecrosis of the femoral head
THA: Total hip arthroplasty
CD: Core decompression
VBG: Vascularized bone graft
NVBG: Non-vascularized bone graft
SCI-EXPANDED: Science Citation Index Expanded
SSCI: Social Sciences Citation Index
